# Inference of epidemiological parameters from household stratified data

**DOI:** 10.1371/journal.pone.0185910

**Published:** 2017-10-18

**Authors:** Camelia R. Walker, Joshua V. Ross, Andrew J. Black

**Affiliations:** 1 Stochastic Modelling and Operations Research Group, School of Mathematical Sciences, University of Adelaide, Adelaide, SA 5005, Australia; 2 ACEMS, School of Mathematical Sciences, University of Adelaide, Adelaide, SA 5005, Australia; University of Florida, UNITED STATES

## Abstract

We consider a continuous-time Markov chain model of SIR disease dynamics with two levels of mixing. For this so-called stochastic households model, we provide two methods for inferring the model parameters—governing within-household transmission, recovery, and between-household transmission—from data of the day upon which each individual became infectious and the household in which each infection occurred, as might be available from First Few Hundred studies. Each method is a form of Bayesian Markov Chain Monte Carlo that allows us to calculate a joint posterior distribution for all parameters and hence the household reproduction number and the early growth rate of the epidemic. The first method performs exact Bayesian inference using a standard data-augmentation approach; the second performs approximate Bayesian inference based on a likelihood approximation derived from branching processes. These methods are compared for computational efficiency and posteriors from each are compared. The branching process is shown to be a good approximation and remains computationally efficient as the amount of data is increased.

## Introduction

First Few Hundred (FF100) studies are data collection exercises carried out in the early stages of pandemic influenza outbreaks [1–4]. The aim of these is to characterise a novel strain to determine its impact and hence inform public health planning [5, 6]. FF100 studies involve the collection of data from households where one person is confirmed to be infected. The members of the household are surveilled to identify their time(s) of symptom onset and the study is continued until the first few hundred cases have been observed, or adequate characterisation has been achieved. Households are the primary unit of observation because they are convenient to surveil—in contrast to more general contact tracing—and a large fraction of transmission occurs within the household [7].

Stochastic models, where the population are split into households with different rates of mixing within and between households, are a natural framework to understand FF100 data [8]. Recent work inferred *within-household* epidemic parameters from this type of household stratified data [9, 10]. In [10], inference is performed using a Bayesian MCMC framework, with exact evaluation of the likelihood, returning a joint posterior distribution for all parameters of interest and hence the within-household reproductive ratio. In this paper we present two methods for performing inference for a Markovian SIR household model—that also infers the between household transmission parameter. The first method uses a standard data-augmented approach [11–13]. The second is a new method based on a branching process approximation, which is potentially more computationally efficient. With an estimate for the between household mixing we can then in turn estimate the household reproductive number, R_*_, and the early growth rate of the epidemic, *r*, which are of importance to public health response [5, 6].

**Fig 1:**
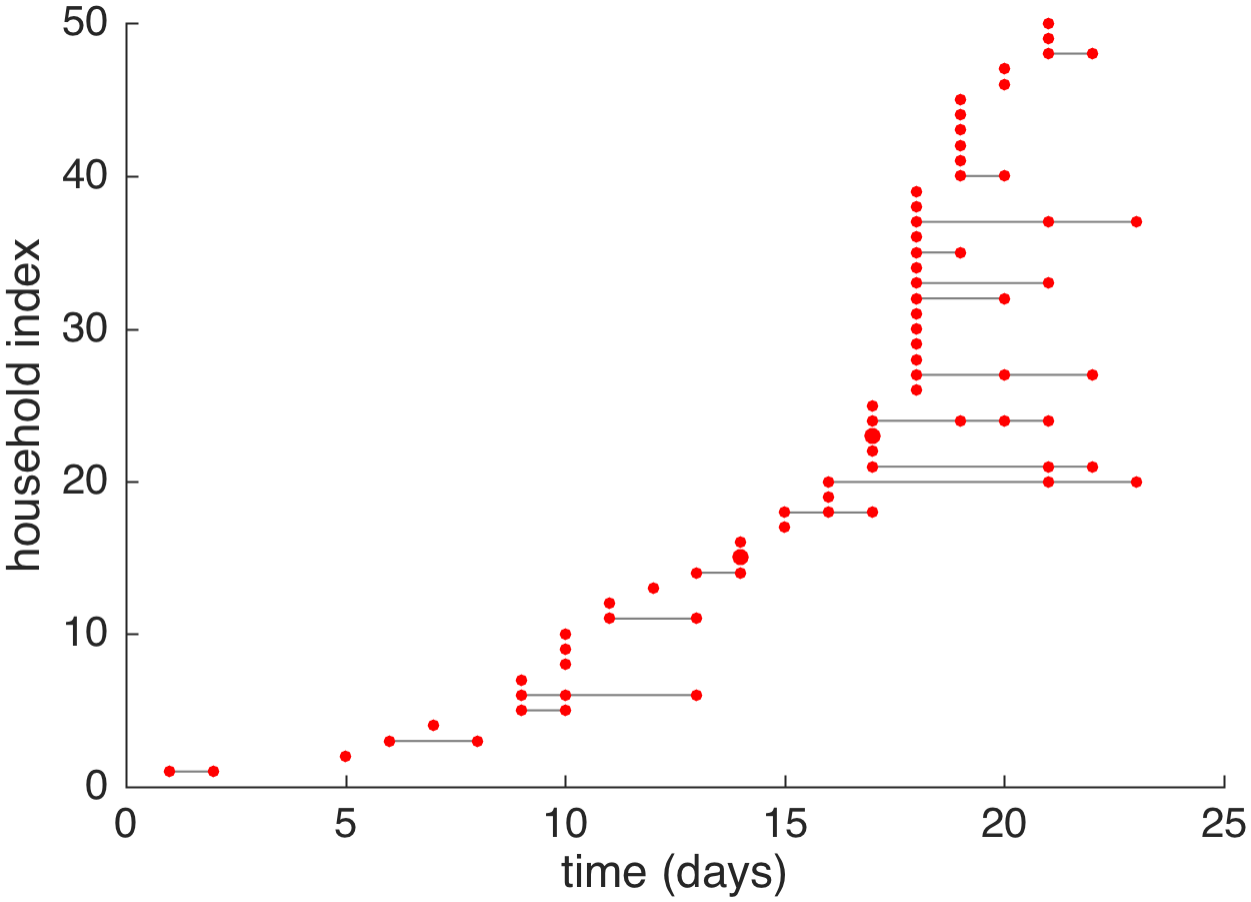
A realisation of the SIR household model. The households are all of size 3 and the model is described in the Models and Methods section. The times of symptom onset, binned into days, in the first 50 infected households at the beginning of an epidemic outbreak are presented. The size of points corresponds to the number of infections on that day. The lines provide a visual reference to link infections within the same household.

The data we assume to be available are illustrated in Fig 1; we observe only the times, at a daily resolution, when individuals become symptomatic, which is assumed to coincide with infectiousness; recovery times are not available. This is realistic for a disease such as influenza where the onset of symptoms and infectiousness are highly correlated, but times of recovery are very hard to identify. The main challenge in this inference problem, as with many similar models and datasets, is that the likelihood is difficult to compute due to the missing data. The standard approach to these sorts of problems is to use a data-augmentation method [11, 14]. In this approach, all unobserved events are treated as unknowns to also be sampled within the MCMC routine; for the model considered in this paper these would be the exact infection and recovery times for each individual within each household. When the exact times are assumed known the likelihood is trivial to evaluate. A data-augmented approach potentially allows great flexibility in model choice and fitting, but the trade off is that the MCMC scheme needed to sample from the joint distribution of parameters and unknown data is quite complex and displays slower mixing. Convergence can be an issue when there is a large amount of missing data [15, 16] and the scalability of these algorithms is poor as more data is added [17]—DA-MCMC is essentially a serial algorithm that works on the whole data set at once and cannot exploit parallelism easily.

Motivated by these problems, we develop another approach, based on approximation of the original process, and compare it to a data-augmentation method. Our main goal in developing this is to produce a more computationally-efficient algorithm that can potentially be used for real-time inference. Our approach is to carefully consider the dynamics and structure of the problem to allow us to derive an approximation to the exact likelihood that can be evaluated using a novel combination of numerical methods (matrix exponential methods [18], stochastic simulations [19] and numerical convolutions). This allows us to use a simple Metropolis-Hastings algorithm to compute a joint posterior for all the parameters of interest. There are three main assumptions underpinning our method. The first is that we can approximate the early time behaviour of the epidemic as a branching process where only a single introduction to each household is possible. This is a very mild assumption and we would expect data collected in the early stages of an outbreak, say from an FF100 study, to conform to this reasonably closely. The second, more technical assumption we make, is that we can replace certain random variables that arise in the problem with their mean values. The third assumption we make is that households are infected at uniformly distributed times on the day of their initial infection. We show that our method provides a good approximation to the full model and the final posteriors that we compute show good convergence to the true model parameters as the amount of household data is increased. The method becomes more efficient than the standard DA-MCMC when dealing with large numbers of households, at the expense of introducing some positive bias in our estimates of the between household transmission rate.

## Models and methods

### Households model and data

The dynamics of the epidemic are modelled as a continuous-time Markov chain. Individuals are grouped into *H* mutually exclusive households and make effective contact at a high rate within households and at a low rate between households. In this paper, for simplicity, we will assume that all households are of the same size, *N*, and an SIR model for disease dynamics. Thus each individual is classified as *susceptible* to infection, *s*, *infectious* and able to infect susceptible individuals, *i*, or *recovered* and immune to the disease, *r*. As *N* is fixed, the state or configuration of a household can be specified by the number of susceptible and infectious individuals within the household (where *r* = *N* − *s* − *i*). Note that in this paper we do not consider models with a latent / exposed period. Extensions allowing for this are detailed in the Discussion.

If we index households by *j* = 1, …, *H*, then the state of the system, *Y*(*t*) can be specified by an *H* × 2 matrix where the *j*’th row gives the number of susceptible and infectious individuals in household *j*,

$$\begin{array}{c} Y\left(t\right)={\left(s_{j}\left(t\right),i_{j}\left(t\right)\right)}_{j=1:H}. \end{array}$$ (1)

Thus the state space is then (dropping the dependence on time),

$$S=\{{\left(s_{j},i_{j}\right)}_{\left(j=1:H\right)}\in {\left\{0,1,\ldots ,N\right\}}^{H\times 2}\;|\;s_{j}+i_{j}\leq N\;\forall \;j\}.$$

Note that there are lower dimensional representations of household models in which a state is a vector which describes the total number of households in each possible configuration [20]; however, we adopt the higher dimensional version here as it simplifies inference.

The dynamics of the SIR household model are defined by the transitions that can occur throughout the population and their corresponding rates. Infectious individuals make effective contact within their household at rate *β*. In household *j* the probability that effective contact within the household leads to an infection is $\frac{s_{j}\left(t\right)}{N-1}$, and hence the rate of within household infection is $\frac{\beta s_{j}\left(t\right)i_{j}\left(t\right)}{N-1}$. Each infectious individual recovers at rate *γ*, so recoveries in household *j* occur at rate *γi*_*j*_(*t*). Lastly, infectious individuals may make effective contact with any individual in the population outside of their own household at rate *α*. Thus between household effective contact results in an infection in household *j* at rate

$$\frac{\alpha s_{j}\left(t\right)\left(I\left(t\right)-i_{j}\left(t\right)\right)}{N(H-1)},$$

where *I*(*t*) = ∑_*j*_ *i*_*j*_(*t*) is the total number of infectious individuals in the population.

We assume that the first infection is seeded in a single household at some U(0, 1) distributed time, *θ*_0_, such that the matrix encoding the first state, *Y*(*θ*_0_), has first row (*N* − 1, 1) and all other rows (*N*, 0). Note that, as our data only reveals cases of infectiousness at a daily resolution, the time of the first infection is unknown.

#### Data

Suppose we have observed the start of an epidemic over some time period (0, *T*]. We assume our data counts the cumulative number of infections in each household each day, where day *t* is defined as the time interval (*t* − 1, *t*]. Here we are assuming that symptoms coincide with infectiousness. Each household is labelled by *j* = 1, …, *M* in the order that they became infected, but note that as the process is only observed at a daily resolution (taken to be the end of each day) the ordering within a day is arbitrary. It is natural to specify this data in terms of two quantities: the days on which each household is infected and the time series of cumulative infection counts within each household, starting from their day of infection. More precisely, let *ψ*_*t*_ be the set of the labels (*j*) of the households that became infected on day *t*. Then let **w**^(*j*)^ = (*w*_*k*_)^(*j*)^ be a vector where *w*_*k*_ is the cumulative number of infection events within the *j*th household, recorded at the end of day *k*, from the day of the households initial infection up to day *T*. Thus the data is completely specified by the sets {*ψ*_*t*_}_*t* = 1: *T*_ and the vectors {**w**^(*j*)^}_*j* = 1: *M*_, which we denote

$$\begin{array}{c} D=\{{\left\{\psi_{t}\right\}}_{t=1:T},{\left\{w^{\left(j\right)}\right\}}_{j=1:M}\}. \end{array}$$ (2)

**Fig 2:**
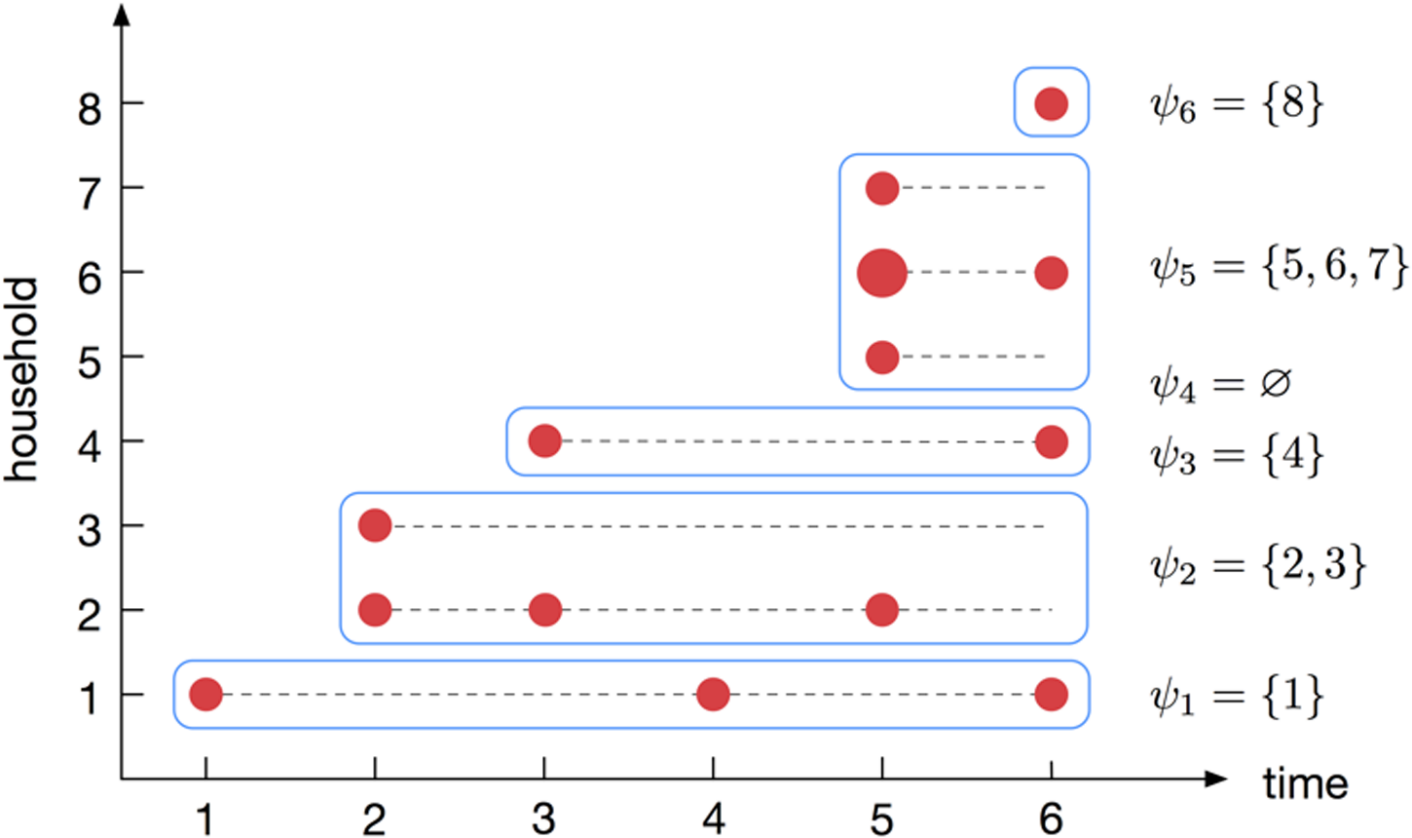
An illustration of how the data is structured for inference. An outbreak observed over *T* = 6 days, resulting in *M* = 8 households becoming infected. The red circles indicate the days on which new infections are observed and their size is proportional to the number of infections. The sets *ψ*_*t*_ indicate which households become infected on day *t*. Note that *ψ*_4_ = ⌀ indicates that no new houses were infected on day 4. The cumulative number of observed cases within each household, over the 6 days are: **w**^(1)^ = (1, 1, 1, 2, 2, 3), **w**^(2)^ = (1, 2, 2, 3, 3), **w**^(3)^ = (1, 1, 1, 1, 1), **w**^(4)^ = (1, 1, 1, 2), **w**^(5)^ = (1, 1), **w**^(6)^ = (2, 3), **w**^(7)^ = (1, 1) and **w**^(8)^ = (1).

These quantities are illustrated for a specific example in Fig 2. We also define $\Omega_{t}=\cup_{j=1}^{t-1}\psi_{j}$, which is the set of labels of households that became infected before day *t*; this will be used in the derivation of the branching process approximation.

### Data augmented MCMC

Data augmented Markov Chain Monte Carlo (DA-MCMC) is a powerful, exact Bayesian inference method for data with missing information. We adopt an approach similar to [11] to infer the joint posterior density of (*α*, *β*, *γ*). The general approach is to construct an augmented likelihood, the joint density of the data and the missing information given the model parameters, and use this to construct a single-component Metropolis-Hastings algorithm. This method proves useful for FF100 study data as the exact times of infection over each day are missing and the number of recovery events, and the times at which they occur, are entirely unknown. Although the data-augmented approach is a standard method for this kind of problem, we are not aware of it having been implemented in a household model where no transition times are known exactly and in which all parameters are unknown. For example, data-augmented MCMC has been implemented for a similar model with data obtained at regular discrete times, however parameters associated with the infectious period or recvery distribution were assumed to be known [12, 13]. Hence we outline the algorithm developed for our particular problem.

As per the usual approach we augment our data with the transition times $\theta \in R^{m}$ and corresponding states **Y** = {*Y*(*θ*_1_), …, *Y*(*θ*_*m*_)} in the underlying model, where *m* is the unknown number of transitions over time (*θ*_0_, *T*] which is allowed to vary. Additionally we consider the classification of infection events as missing, that is, we augment the data by transition labels *ζ* ∈ {recovered, within, between}^*m*^. This is such that we can construct sets of transition indices, *A*, *B* and *C*, which correspond to within-household infection, between-household infection and recovery events respectively. In writing down the expression for the augmented likelihood function we adopt the convention that all quantities (*s*, *i*) are evaluated immediately prior to a transition. Hence we have,

$$\begin{array}{ccc} L_{DA} & ≔ & f(D,\theta ,Y,\zeta |\alpha ,\beta ,\gamma ,\theta_{0})\\ & = & 1_{\left\{D,\theta ,Y,\zeta \right\}}\;\prod_{j\in A}\;\frac{\beta s^{\left(j\right)}i^{\left(j\right)}}{N-1}\;\prod_{k\in B}\;\frac{\alpha s^{\left(k\right)}(I^{\left(k\right)}-i^{\left(k\right)})}{N(M-1)}\;\prod_{l\in C}\;\gamma i^{\left(l\right)}\\ & & \times \text{exp}\;\{-\sum_{p=1}^{m+1}\sum_{c=1}^{H}\;(\frac{\beta s_{c}^{\left(p\right)}i_{c}^{\left(p\right)}}{N-1}+\frac{\alpha s_{c}^{\left(p\right)}(I^{\left(p\right)}-i_{c}^{\left(p\right)})}{N(M-1)}+\gamma i_{c}^{\left(p\right)})\;\left(\theta_{p}-\theta_{p-1}\right)\}, \end{array}$$

where superscripts denote transition indices, subscripts denote household indices, terms without a subscript refer to the household which changes state, $1_{\left\{D,\theta ,Y,\zeta \right\}}$ denotes an indicator function corresponding to one if the data, D, could have arisen from the events defined by (***θ***, **Y**, *ζ*) and *θ*_*m*+1_ ≔ *T* for simplicity. The indicator function ensures that the augmented data has the same number of infections in each household, each day, as our observed data, and that the augmented data corresponds to a feasible realisation of a household SIR model. For example, the indicator takes the value 0 if there is a within-household infection in a completely susceptible household. Note that inference could be made without labelling the two kinds of infection, however this more explicit representation produces gamma or truncated gamma marginal densities of *β* and *α* for uniform, gamma, inverse uniform or truncated gamma priors; hence they may be efficiently sampled.

Marginal posterior densities of *α*, *β*, *γ* and *θ*_0_ can be evaluated and sampled from in a similar way to [11]. Lastly the joint posterior density, $f(\theta ,Y,\zeta |\beta ,\gamma ,\theta_{0},D)$, is proportional to *L*_*DA*_, thus it can be sampled from by randomly choosing from the following five kinds of moves according to an arbitrary probability mass function with non-zero components, {*q*_1_, …, *q*_5_}:

(i) Randomly select an infection time, *θ*_*j*_, choose a candidate Uniform(⌊*θ*_*j*_⌋, ⌈*θ*_*j*_⌉) distributed infection time, where ⌊⋅⌋ and ⌈⋅⌉ refer to the floor and ceiling function respectively. Let the augmented likelihood corresponding to the candidate be denoted by ${\hat{L}}_{DA}$. The new point is accepted with probability
  $$\begin{array}{c} \text{min}\;\{\frac{{\hat{L}}_{DA}}{L_{DA}},1\}; \end{array}$$ (3)
(ii) Randomly select an infection event and change its type, *ζ*^(*j*)^, from *between* to *within* household infection or vice versa. The new point is accepted with probability as in Eq (3);
(iii) Randomly select a recovery time, *θ*_*j*_, and choose a candidate Uniform(*θ*_*k*_, *T*) distributed recovery time, where *θ*_*k*_ is the time of the first infection within the household. The new point is accepted with probability as in Eq (3);
(iv) Insert a Uniform(*θ*_*k*_, *T*) distributed recovery time in a randomly chosen household. Let *M* be the number of households infected by time *T* and |*C*| be the number of recovery transitions in **Y**. The new point is accepted with probability
  $$\text{min}\;\{\frac{{\hat{L}}_{DA}M\;(T-\theta_{k})\;q_{5}}{L_{DA}\left(|C|+1\right)q_{4}},1\};\;\text{or},$$
(v) Randomly select and remove a recovery event with probability
  $$\text{min}\;\{\frac{{\hat{L}}_{DA}\left|C\right|q_{4}}{L_{DA}M\;(T-\theta_{k})\;q_{5}},1\}.$$

Acceptance probabilities are the ratio of the likelihood functions multiplied by the probability of returning to the current state, divided by the proposal density. For moves (iv) and (v), related to the insertion and removal of recovery times, we have that the probability density of choosing move (iv), selecting a particular household and inserting a recovery time is *q*_4_/(*M*(*T* − *θ*_*k*_)), and the probability of choosing move (v) and selecting one of |*C*| recoveries is *q*_5_/|*C*|.

Each iteration of the DA-MCMC algorithm is comprised of Gibbs samples of *α*, *β*, *γ* and *θ*_0_ followed by a Hastings step for (***θ***, **Y**, *ζ*) as per (i)-(v). The distribution of these samples converge to the joint posterior distribution of (***θ***, **Y**, *ζ*, *α*, *β*, *γ*), though consecutive samples will be highly correlated. The marginal over the parameters is simply obtained by ignoring the samples of (***θ***, **Y**, *ζ*).

### Branching process approximation

We now provide an approach for analysing the household model by approximating it by a model that acts like a branching process at the household level. This model is equivalent to the model obtained by letting the number of households tend to infinity. As a consequence, between-household infection occurs into completely susceptible households almost surely; this approximation is reasonable, as the data we wish to perform inference on is from the very earliest stages of an outbreak in a large population. The main reason for considering the branching process model is that households act independently after initial infection. Hence we can consider the dynamics within each infected household, following their initial infection, in isolation from each other [8, 21]. Under this model we construct an approximate likelihood with the aim of obtaining accurate estimates for the joint posterior distribution of (*α*, *β*, *γ*). The posteriors using this approximation are compared with the full household epidemic model in the Results section. We show that the resulting posterior distribution approximates the exact posterior distribution of the household model well, while the independence assumption allows for computational gains in the inference as the data set grows in size.

As we are considering households in isolation from each other, we define the state space for a single household as

$$\begin{array}{c} S=\{\left(s,i\right)\in {\left\{0,1,...,N\right\}}^{2}:s+i\leq N\}. \end{array}$$

The within-household dynamics are defined by the transitions that can occur within an individual household and their corresponding rates; these are simply the within-household infection and recovery transitions with rates as described before. The within-household process can be defined in terms of its infinitesimal transition rate matrix, *Q*, given by

$${\left[Q\right]}_{f(s,i),f(x,y)}=\{\begin{array}{cc} \frac{\beta si}{N-1} & \text{for}\;(x,y)=(s-1,i+1)\text{,}\;s\geq 1\\ \gamma i & \text{for}\;(x,y)=(s,i-1)\text{,}\;i\geq 1\\ -\frac{\beta si}{N-1}-\gamma i & \text{for}\;(x,y)=(s,i)\\ 0 & \text{otherwise,} \end{array}$$

where f:S→{1,...,|S|} is a bijective map [22]. The first infection within each household moves it into state (*N* − 1, 1), at which point the within-household dynamics determine how the disease spreads within the household for the remainder of the epidemic.

We assume that between-household infection occurs due to homogeneous mixing of all the individuals in the population at rate *α*, thus the rate at which new households are infected is simply *αI*(*t*), where *I*(*t*) is the total number of infected individuals in the population at time *t*. The model is initialised with a single infected household at a U(0, 1) distributed time.

For this model we can identify the threshold parameter, R_*_, which is a household (population level) reproduction number [8]. This is the expected number of households infected by a primary infectious household in an otherwise susceptible population of households; where a household is considered infectious while it contains at least one infectious individual and a household is considered susceptible if it contains only susceptible individuals. It is one of at least five reproductive numbers that might be used when assessing the controllability of a disease in a community of households [23–25], but we adopt it herein as it is relatively easy to calculate and interpret. Let ${\left\{X_{t}\right\}}_{t\in R^{+}}$ be the Markovian process that describes the state of an individual household from the time of its infection (i.e., the time of the first infection within the household). Let *I*(*k*) be the function which returns the number of infectious individuals corresponding to state *k*. Then we have

$$\begin{array}{c} R_{*}=E\;[\int_{0}^{\infty }\;\alpha I\left(X_{t}\right)\;dt], \end{array}$$

where *X*_0_ = (*N* − 1, 1) is the initial state of the process [8, 26, 27]. This can be calculated by solving a system of linear equations that depend on the parameters of the epidemic model [27, 28].

Also of interest from a public health perspective, is the early growth rate, *r*; this is also called the Malthusian parameter. Under the same conditions as above, this is defined as the unique solution to

$$\begin{array}{c} E\;[\int_{0}^{\infty }\;\alpha I\left(X_{t}\right)e^{-rt}\;dt]=1. \end{array}$$

This can once again be evaluated efficiently [27].

#### Approximate likelihood

The branching process likelihood approximation relies on expressing the likelihood in terms of the data on a given day, *t*, partitioned into newly infected households, *ψ*_*t*_, and formerly infected households each day, $\Omega_{t}=\cup_{j=1}^{t-1}\psi_{j}$ (see Fig 2 for an example). With this partition, the likelihood for (*α*, *β*, *γ*) can then be written as,

$$\begin{array}{c} L\left(\alpha ,\beta ,\gamma \right)=\prod_{t=1}^{T}\;P\;({\left\{w^{\left(j\right)}\right\}}_{j\in \psi_{t}}|\psi_{t})\;P\;(\psi_{t}|{\left\{w^{\left(j\right)}\right\}}_{j\in \Omega_{t}}); \end{array}$$ (4)

we have invoked the independence between ({**w**^(*j*)^}_*j*∈*ψ*_*t*__|*ψ*_*t*_) and {**w**^(*j*)^}_*j*∈Ω_*t*__ due to the branching process assumption. That is, we use the fact that under the branching process assumption households are conditionally independent given their initial infection. Note that we have split the likelihood in a way that does not use the Markov property, this is because the Markov property can not be easily exploited here as the state of the process is never observed exactly.

As Ω_1_ = ⌀, that is, there are no households infected before *t* = 0, the term

$$\begin{array}{c} P\;(\psi_{1}|{\left\{w^{\left(j\right)}\right\}}_{j\in \Omega_{1}})=P\;(\psi_{1}) \end{array}$$

is determined by the initial condition. Further, households in *ψ*_*t*_ are identically distributed in the absence of within-household information. Thus their labels are arbitrary and only the number of households in *ψ*_*t*_ is relevant, that is

$$P\;(\psi_{t}|{\left\{w^{\left(j\right)}\right\}}_{j\in \Omega_{t}})=P\;(|\psi_{t}\left|\right|{\left\{w^{\left(j\right)}\right\}}_{j\in \Omega_{t}}),$$

where |*ψ*_*t*_| denotes the set norm of *ψ*_*t*_, that is, the number of households infected over day *t*. Thus we can factor the likelihood, Eq (4), into two parts that are related to the within-household dynamics and between-household dynamics respectively. That is,

$$L\left(\alpha ,\beta ,\gamma \right)=L_{w}\left(\alpha ,\beta ,\gamma \right)L_{b}\left(\alpha ,\beta ,\gamma \right),$$

where

$$\begin{array}{c} L_{w}\left(\alpha ,\beta ,\gamma \right)=\prod_{t=1}^{T}\;P\;({\left\{w^{\left(j\right)}\right\}}_{j\in \psi_{t}}|\psi_{t}) \end{array}$$ (5)

and

$$\begin{array}{c} L_{b}\left(\alpha ,\beta ,\gamma \right)=\prod_{t=1}^{T}\;P\;(|\psi_{t}\left|\right|{\left\{w^{\left(j\right)}\right\}}_{j\in \Omega_{t}}). \end{array}$$ (6)

We refer to *L*_*w*_ as the within-household likelihood function and *L*_*b*_ as the between-household likelihood function. In the following we detail how we calculate *L*_*b*_ and *L*_*w*_.

#### Between-household likelihood, *L*_*b*_

Each term in the product for the between-household likelihood, Eq (6), is the probability that we observe *H*_*t*_ ≔ |*ψ*_*t*_| new infected households on day *t*, given the data, over the time period [0, *T*], for households that were infected before day *t*. We decompose *H*_*t*_ into two components, $H_{t}^{\left(1\right)}$ and $H_{t}^{\left(c\right)}$, such that $H_{t}=H_{t}^{\left(1\right)}+H_{t}^{\left(c\right)}$. The first component, $H_{t}^{\left(1\right)}$, is the number of the newly infected households on day *t* that are infected by a household in Ω_*t*_, i.e., a household infected before day *t*. The second component, $H_{t}^{\left(c\right)}$, is the remaining number of newly infected households on day *t*, i.e., those that are infected by households that become infected on day *t*. We do not observe this demarcation in our data, but it assists us in the evaluation of the likelihood.

We start by considering the calculation of the probability mass function (pmf) of $H_{t}^{\left(1\right)}$, denoted $h_{t}^{\left(1\right)}$. Then, we consider the evaluation of the pmf of $H_{t}^{\left(c\right)}$, $h_{t}^{\left(c\right)}$. The required pmf of *H*_*t*_, **h**_*t*_, is subsequently evaluated using efficient methods for calculating convolutions.

#### First generation of households, $H_{t}^{\left(1\right)}$

To calculate $h_{t}^{\left(1\right)}$, the pmf of the number of first generation infected households, we note that on the first day of the epidemic there is only a single household infected at a *U*(0, 1) distributed time. Hence there is exactly 1 infected household in the first generation of households, so

$$P\;(H_{1}^{\left(1\right)}=1)=1.$$

For *t* ≥ 2 we consider the rate at which the households in Ω_*t*_ infect new households. As we model the outbreak as a branching process, we assume that only completely susceptible households are infected, hence the instantaneous rate of infection at time *τ* ∈ (*t* − 1, *t*] from the households in Ω_*t*_ is

$$\begin{array}{c} \alpha \sum_{j\in \Omega_{t}}I\;(X_{\tau }^{j}), \end{array}$$

where $X_{\tau }^{j}$ is the state of household *j* at time *τ* and *I*(*k*) is a function returning the number of infectious individuals in a household in state *k*.

Thus the first generation of households are created as an inhomogeneous Poisson process, and conditioning on the information about the households in Ω_*t*_, {**w**^(*j*)^}_*j* ∈ Ω_*t*__, we have

$$\begin{array}{c} H_{t}^{\left(1\right)}|{\left\{w^{\left(j\right)}\right\}}_{j\in \Omega_{t}}∼\text{Poisson}\;(\alpha \sum_{j\in \Omega_{t}}\int_{t-1}^{t}I\;(X_{\tau }^{j}|w^{\left(j\right)})\;d\tau ). \end{array}$$ (7)

Hence, we need to evaluate the distribution of

$$\begin{array}{c} \Lambda_{t}≔\alpha \sum_{j\in \Omega_{t}}\int_{t-1}^{t}I\;(X_{\tau }^{j}|w^{\left(j\right)})\;d\tau . \end{array}$$

However, this is expensive to compute, so instead we replace Λ_*t*_ in Eq (7) with its expectation, which can be evaluated in a feasible manner. Precisely, we use

$$\begin{array}{c} P\;(H_{t}^{\left(1\right)}=h|{\left\{w^{\left(j\right)}\right\}}_{j\in \Omega_{t}})\approx \frac{e^{-E[\Lambda_{t}]}E{\left[\Lambda_{t}\right]}^{h}}{h!}, \end{array}$$

where

$$\begin{array}{c} E\left[\Lambda_{t}\right]=\alpha \sum_{j\in \Omega_{t}}\int_{t-1}^{t}E\;[I\;(X_{\tau }^{j}|w^{\left(j\right)})]\;d\tau . \end{array}$$ (8)

This approximation allows tractability of the between-household likelihood; we do not however restrict the paths of households in Ω_*t*_ such that Λ_*t*_ = *E*[Λ_*t*_]. As Λ_*t*_ is the force of infection over a short time period (a day) it should have relatively low variance, thus replacing Λ_*t*_ by its expectation may provide a reasonable approximation. Later we detail how the conditional expectations, Eq (8), can be calculated using matrix exponential methods.

#### Subsequent generations of households, $H_{t}^{\left(c\right)}$

Recall that the number of newly infected households on day *t* is $H_{t}=H_{t}^{\left(1\right)}+H_{t}^{\left(c\right)}$. The first component, $H_{t}^{\left(1\right)}$, is the number of the newly infected households on day *t* that are infected by a household in Ω_*t*_, i.e., a household infected before day *t*. The second component, $H_{t}^{\left(c\right)}$, is the remaining number of newly infected households on day *t*, i.e., those that are infected by households that become infected on day *t*.

We assume that the infection of the $H_{t}^{\left(1\right)}$ households are uniformly distributed over day *t*, and since their dynamics are independent, we have that $H_{t}^{\left(c\right)}$ is the convolution of $H_{t}^{\left(1\right)}$ random variables; we will use *G* to denote one of these random variables. Each of these random variables correspond to the size of a household branching process at time 1 that was initialised at a Uniform(0, 1) time. The calculation of the pmf of *G* is once again computationally expensive, so here we choose to estimate this distribution using simulation [19]. We are simulating over a short period of time (a day) and use the most efficient representation to minimise computational time. This allows for a large number of simulations to be produced in a computationally-efficient manner.

Once we have estimated the pmf of *G*, we can calculate **h**_*t*_ from $h_{t}^{\left(1\right)}$ as

$$\begin{array}{c} h_{t}=Mh_{t}^{\left(1\right)}, \end{array}$$

where the convolution matrix, M, is defined as follows. Let *ϕ* be a column vector of the pmf of the random variable *G* + 1. Then let

$$\begin{array}{c} c_{j}=c_{j-1}*\phi ,\;j\geq 2, \end{array}$$

where ‘*’ denotes a discrete convolution and **c**_1_ = *ϕ*. The matrix M is then given by

$$\begin{array}{c} M=[e_{1},c_{1},c_{2},\ldots ], \end{array}$$

where **e**_1_ is a vector of 0s with the exception of a 1 in the first entry. The matrix *M* is truncated such that no probability needed for the calculation of the likelihood is lost. The calculation of this is not expensive, even for large matrices as the convolutions can be done using discrete Fourier transforms [29]. In this paper we simply use the built in MATLAB function conv(), although other methods may provide computational gains, if required.

#### Single household dynamics, *E*[Λ_*t*_] and *L*_*w*_

Recall, the evaluation of the pmf $h_{t}^{\left(1\right)}$ for *t* ≥ 2, corresponding to the number of first generation infected households on day *t*, requires the evaluation of the expected force of infection over day *t* from households infected prior to day *t*, *E*[Λ_*t*_]. We begin by detailing the evaluation of *E*[Λ_*t*_], and then note how the within-household likelihood, *L*_*w*_, follows.

The computation of the *E*[Λ_*t*_] can be expressed in terms of integrals of the expected number of infectious individuals within each household in Ω_*t*_, Eq (8). As this expression is a sum over independent households we simplify our exposition, by detailing the calculation for a single arbitrary household in Ω_*t*_, with observed data **w** (thus dropping the superscript ‘*j*’ notation for now). The independence also means we can rescale time within the household to begin at the start of the day of the first infection. Thus we need to calculate the expected number of infected individuals over each of the |**w**| = *ω* days since the first infection within that house, i.e.

$$\begin{array}{cc} E\;[I\;(X_{\tau }|w)] & =\sum_{k\in S}\;I\;\left(k\right)P\;(X_{\tau }=k|w), \end{array}$$ (9)

for all *τ* ∈ (*t* − 1, *t*], where *t* = 2, …, *ω*. Note that as we are conditioning on the entire observed data within the household, **w**, the random variable *ω* is implicitly conditioned on. That is, we are conditioning on knowing that the household became infected on day *T* − *ω* + 1. In the remainder of this subsection all probabilities are conditioned on *ω*, but this is not written explicitly for concision.

The expectation Eq (9) can be calculated efficiently, and hence the integral of the expectation to find the force of infection can also be calculated efficiently and accurately with Simpsons Rule, say. Our calculation is similar to that of the forward-backward algorithm [30], but is more involved as we need to calculate the expectations for all *τ*, not just the discrete time points at which observations occur. First we define some quantities. As *w*_*t*_ is the total number of infections observed in the household by the end of day *t* (within-household time), *X*_*t*_ must be in a set of states such that *N* − *s*(*t*) = *w*_*t*_. These states are encoded by indicator vectors, **z**_*t*_, with 1s in entries corresponding to states where *N* − *s*(*t*) = *w*_*t*_ and zeros otherwise; these are either row or column vectors as required.

Define the row vector **f**_*t*_ as the ‘forward’ probabilities of the system, so the *k*th element is the probability the system is in state *k* at the end of day *t*, given the observed data up to *t*,

$$\begin{array}{c} {\left[f_{t}\right]}_{k}=P\;(X_{t}=k|w_{\left(1:t\right)}). \end{array}$$

These can be calculated in a recursive manner as follows:

$$\begin{array}{c} f_{t}=\frac{\left(f_{t-1}e^{Q}\right)\circ z_{t}}{\left(f_{t-1}e^{Q}\right)\cdot z_{t}},\;t=2,\ldots ,\omega , \end{array}$$

where ‘∘’ is an element-wise vector product. The first vector, **f**_1_, is determined from the initial condition as follows: let **v** be a probability vector with a 1 in the entry corresponding to state (*N* − 1, 1). Then, as the infection is introduced into each household at a Uniform(0, 1) distributed time on their day of infection, the distribution of *X*_1_ is given by

$$\begin{array}{c} u=\int_{0}^{1}\;ve^{Q(1-\tau )}d\tau . \end{array}$$

Conditioning on *w*_1_ gives **f**_1_ = **u** ∘ **z**_1_/**u** ⋅ **z**_1_.

We also define the ‘backward’ probabilities, **b**_*t*_, with elements

$${\left[b_{t}\right]}_{k}=P\;(w_{\left(t+1:\omega \right)}|X_{t}=k).$$

These are the probabilities of observing the remainder of the data given that the system is in state *k* at the end of day *t*. These can be calculated in a similar recursive way to the forward probabilities, but working backward from the final observation:

$$\begin{array}{c} b_{t-1}=e^{Q}\left(b_{t}\circ z_{t}\right),\;t=\omega ,\ldots ,2, \end{array}$$

with **b**_*ω*_ = **1**.

Applying Bayes’ theorem to the pmf in Eq (9) and using the Markov property we arrive at

$$\begin{array}{c} P\;(X_{\tau }=k|w)=\frac{P\;(X_{\tau }=k|w_{\left(1:t-1\right)})\;P\;(w_{\left(t:\omega \right)}|X_{\tau }=k)}{P\;(w_{\left(t:\omega \right)}|w_{\left(1:t-1\right)})}, \end{array}$$ (10)

for *τ* ∈ (*t* − 1, *t*]. Using the law of total probability and the Markov property on the three probability expressions in Eq (10) gives

$$P\;(X_{\tau }=k|w_{\left(1:t-1\right)})={\left[f_{t-1}e^{Q(\tau -t+1)}\right]}_{k},$$

$$P\;(w_{\left(t:\omega \right)}|X_{\tau }=k)={\left[e^{Q(t-\tau )}\left(b_{t}\circ z_{t}\right)\right]}_{k},$$

and

$$P\;(w_{\left(t:\omega \right)}|w_{\left(1:t-1\right)})=f_{t-1}\cdot b_{t-1}.$$

Hence Eq (9) can be expressed in a vectorised form as

$$\begin{array}{c} E\;[I\;(X_{\tau }|w)]=i\;\cdot \;(\frac{f_{t-1}e^{Q(\tau -t+1)}\circ e^{Q(t-\tau )}\left(b_{t}\circ z_{t}\right)}{f_{t-1}\cdot b_{t-1}}),\;t=2,\ldots ,\omega , \end{array}$$

where **i** is a vector whose elements are the number of infected individuals in each state.

This allows us to numerically evaluate $h_{t}^{\left(1\right)}$; note that all matrix exponential calculations here can be expressed as [*e*^*Q*^]^*a*^, so we only need to compute the matrix exponential once per parameter set and take powers of the resulting matrix. Further, when numerically integrating, using a symmetric grid about *t* − 1/2 allows us to take advantage of the symmetry of *e*^*Q*(*τ*−*t*+1)^ and *e*^*Q*(*t*−*τ*)^, effectively halving the number of times we need to take powers of *e*^*Q*^.

Using the quantities calculated above, we can also calculate the within-household likelihood, *L*_*w*_, described in Eq (5). Let *l*^(*j*)^ denote the length of **w**^(*j*)^. Note, under the branching process assumption, infected households act independently of each other, so their within household dynamics following their infection are independent. Hence,

$$\begin{array}{ccc} L_{w}\left(\alpha ,\beta ,\gamma \right) & = & \prod_{t=1}^{T}\;P\;({\left\{w^{\left(j\right)}\right\}}_{j\in \psi_{t}}|\psi_{t})\\ & = & \prod_{t=1}^{T}\;\prod_{j\in \psi_{t}}\;P\;(w^{\left(j\right)}|j\in \psi_{t})\\ & = & \prod_{j=1}^{M}\;P\;(w^{\left(j\right)}|l^{\left(j\right)}). \end{array}$$

Let $f_{t}^{\left(j\right)}$ and $z_{t}^{\left(j\right)}$ denote the forward probability, and the state indicator vector on day *t* for household *j*, respectively. The probability of observing the data in each household is

$$\begin{array}{ccc} P\;(w^{\left(j\right)}|l^{\left(j\right)}) & = & P\;(w_{1})\;\prod_{t=2}^{l^{\left(j\right)}}\;P\;(w_{t}|w_{\left(1:t-1\right)})\\ & = & (u\cdot z_{1}^{\left(j\right)})\;\prod_{t=2}^{l^{\left(j\right)}}\;(f_{t-1}^{\left(j\right)}e^{Q})\;\cdot \;z_{t}^{\left(j\right)}, \end{array}$$

that is, the within-household likelihood is a product of the normalising constants for the forward probabilities. Thus the within-household likelihood is calculated as a by-product of the expectation calculations.

## Results

Our inference methods are compared based upon 50 simulations with true parameter values (*α*, *β*, *γ*) = (0.32, 0.4, 1/3) and 50,000 households of size *N* = 3 (the average household size in Australia is estimated to be 2.6 [31]). These simulations are from the full stochastic household model, not the simplified model where households are conditionally independent after their initial infection. These parameters are chosen such that the average infectious period is three days (this is a typical infectious period for influenza), *R*_0_ = *β*/*γ* = 1.2 and R_*_ ≈ 1.8. For the branching process approximation (BPA) the number of realisations used to estimate the distribution of *G* was 10^3^.

**Fig 3:**
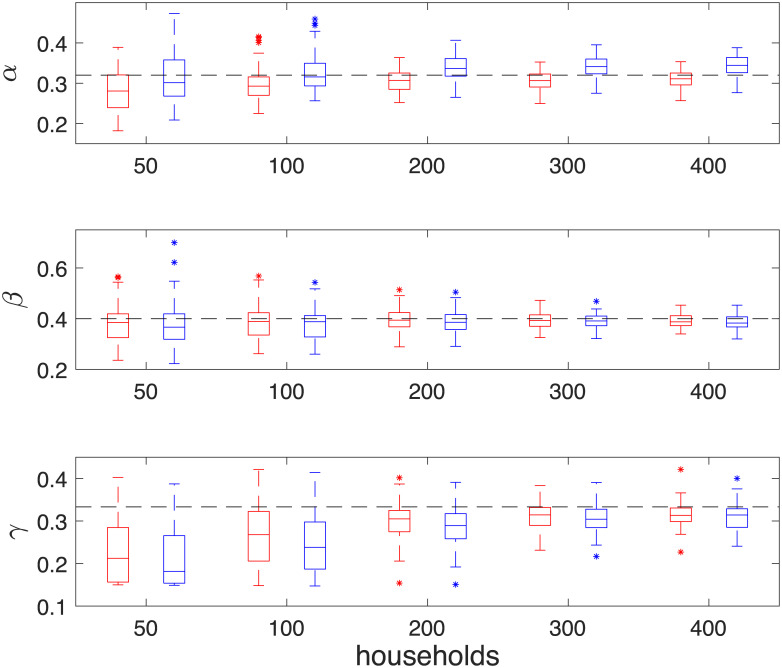
Boxplots of maximum a posteriori (MAP) estimates of (*α*, *β*, *γ*) from 50 simulations. Red and Blue boxes correspond to results from 2.5 × 10^6^ iterations, thinned to 2.5 × 10^5^ samples, of the DA-MCMC algorithm and 10^5^ iterations of the BPA and respectively. MAP’s are calculated from 3 dimensional kernel density estimates. The pairs of boxes from left to right are MAP’s from inference based upon data with 50, 100, 200, 300 and 400 infected households. Black dotted lines indicate the true parameter values at (*α*, *β*, *γ*) = (0.32, 0.4, 1/3).

**Fig 4:**
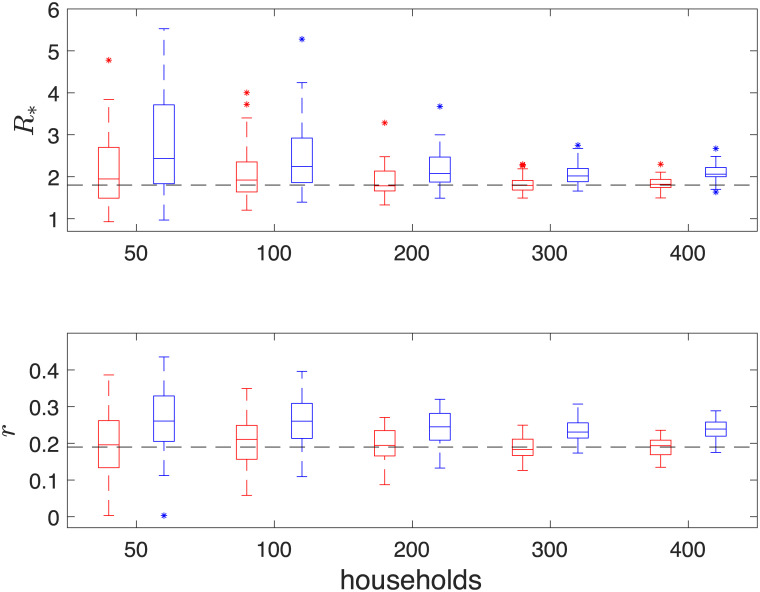
Boxplots of maximum a posteriori (MAP) estimates of (R_*_, *r*) from 50 simulations. Red and Blue boxes correspond to results from 2.5 × 10^6^ iterations (thinned to 2.5 × 10^5^) of the DA-MCMC algorithm and 10^5^ iterations of the BPA and respectively. MAP’s are calculated from 2 dimensional kernel density estimates. The pairs of boxes from left to right are MAP’s from inference based upon data with 50, 100, 200, 300 and 400 infected households. Black dotted lines indicate the true parameter values at (R_*_, *r*) ≈ (1.803, 0.190).

**Fig 5:**
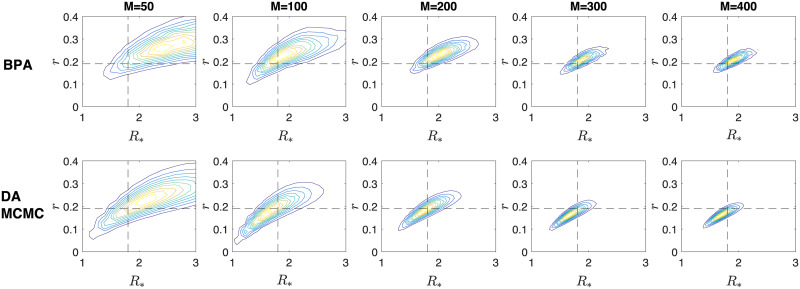
Contour plots of the joint posterior density of R_*_ and *r* from a single simulation. The top and bottom panels are results from 10^5^ iterations of the BPA and 2.5 × 10^6^ iterations, thinned to 2.5 × 10^5^ samples, of the DA-MCMC algorithm respectively. The panels from left to right are posteriors from inference based upon data with 50, 100, 200, 300 and 400 infected households. The intersection of the black dotted lines indicate the true parameter values at (R_*_, *r*) ≈ (1.803, 0.190).

Each algorithm is based upon a Bayesian Markov chain Monte Carlo (MCMC) framework in order to estimate the joint posterior distribution of our parameters [32]. In particular, the BPA is a Metropolis-Hastings algorithm and the DA-MCMC is a single-component Metropolis-Hastings algorithm. Each algorithm is run at various stages of the epidemic in order to show how the posterior distributions converge as more households become infected; the inference for each simulation is run after 50, 100, 200, 300 and 400 households become infected. For the BPA, for each simulation, at each stage of the epidemic, 10^5^ MCMC samples are obtained with a burn-in of 1000 iterations. For the DA-MCMC, for each simulation, at each stage of the epidemic, 2.5 × 10^6^ iterations are run with an additional burn-in of 10^6^ iterations and results are thinned to a sample of size 2.5 × 10^5^. These numbers of iterations were chosen so that each sample had approximately the same effective sample size (ESS). More iterations are needed for the DA-MCMC as the mixing is slower, the samples were thinned for data storage reasons. Both algorithms are implemented with prior distribution for $\left(\alpha ,\frac{\beta }{\gamma },\frac{1}{\gamma }\right)$ of *U*(0.05, 1) × *U*(0.25, 4) × *U*(0.25, 7). The BPA was implemented with a

$$\begin{array}{c} X|Y∼N\;(Y,\;[\begin{array}{ccc} 0.01 & 0 & 0\\ 0 & 0.02 & 0\\ 0 & 0 & 0.05 \end{array}]) \end{array}$$

proposal distribution. The DA-MCMC is implemented by proposing moves (i)-(v) with probabilities *q*_1_ = *q*_2_ = 0.05 and *q*_3_ = *q*_4_ = *q*_5_ = 0.3 respectively. Our results are displayed in terms of maximum a posteriori (MAP) estimates of the model parameters, (*α*, *β*, *γ*), in Fig 3, and MAP estimates of key epidemiological parameters (R_*_, *r*), in Fig 4, and joint posterior density estimates of (R_*_, *r*), in Fig 5. All kernel densities were estimated using the freely available MATLAB packages kde2.m and akde.m [33]. Means and standard deviations for MAP estimates are given explicitly in Table 1.

**Table 1:** Means and standard deviations of maximum a posteriori (MAP) estimates of (*α*, *β*, *γ*, R_*_, *r*). The means and standard deviations of the 50 MAP estimates based upon data with 400 infected households for each parameter is shown in the form mean(standard deviation) for the BPA and DA-MCMC methods. The last row shows the difference in the mean and standard deviation between the two methods.

	*α*	*β*	*γ*	R_*_	*r*
True Values	0.32	0.4	0.333	1.803	0.190
BPA	0.345(0.028)	0.387(0.027)	0.310(0.034)	2.091(0.210)	0.237(0.028)
DA	0.312(0.022)	0.392(0.027)	0.314(0.032)	1.839(0.168)	0.191(0.024)
BPA-DA	0.033(0.012)	-0.005(0.012)	-0.004(0.020)	0.2519(0.066)	0.046(0.008)

From Fig 3, we observe that MAP estimates begin negatively biased for all parameters and converge towards fixed points as more data is obtained. The median of the MAPs of the BPA method for *β* and *γ* are lower than that of the DA-MCMC method, whereas the median of the MAPs of *α* are higher. The boxes associated with *β* and *γ* for each method are overlapping, whereas the boxes associated with *α* are biased higher for the BPA method when data is based upon 300 and 400 infected households. In Fig 4, we observe that the boxes of the MAP estimates converge to the true values of R_*_ and *r* for the DA-MCMC method, whereas they are biased above the true value for the BPA method. The positive bias in these quantities is due to the overestimation of *α* by the BPA method. The box plots indicate a general trend that the variability of the MAP estimates decrease as more data is obtained. It should be noted that these box plots do not show the correlation structure of the parameters; this is not presented here as the dimension of the parameter space makes the correlation structure difficult to display. In Fig 5 the posterior densities of R_*_ and *r* appear similar between the two methods, although the bias of the BPA is clear.

For both methods the variability in the posterior distribution is observed to decrease in a similar way as more households are infected. Table 1 shows that when inference is run after 400 households are infected, the mean of the MAPs of both methods lie within a standard deviation from the true values of (*α*, *β*, *γ*). We also find that the average MAP estimates of $\frac{\beta }{\gamma }$ is found to be 1.2484 and 1.2484 in both methods; this excellent agreement at the household level indicates that the branching process is an appropriate approximation for the full household epidemic process. Out of the two methods, only the means of the MAPs from the DA-MCMC method lie within a standard deviation of the true values of R_*_ and *r*. As the DA-MCMC method is an exact method, and both methods were run on the same simulations, we can compare the difference of MAPs from the two methods, this is given in the final row of Table 1. The difference of the MAPs for *β* and *γ* lie within a standard deviation of 0, the difference for *α*, R_*_ and *r* are in excess of 2.5 standard deviations from 0. This indicates that the BPA method leads to a significantly different answer, in terms of *α*, R_*_ and *r* compared to exact methods. On average we saw a 7.8%, 16.0% and 24.7% positive error in *α*, R_*_ and *r* respectively.

**Fig 6:**
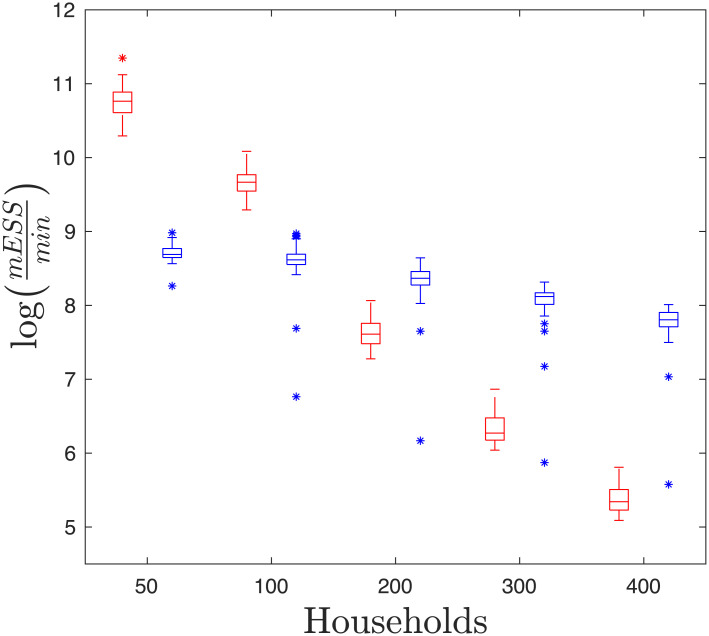
Boxplots of the efficiency of each method against the number of infected households. Here efficiency is presented in terms of log multivariate effective sample size per hour. These estimates are based upon running each algorithm for a 50 simulations with 50, 100, 200, 300 and 400 infected households.

The efficiency of the two algorithms cannot be compared directly in terms of iterations per time, as samples from the DA-MCMC are more highly correlated than samples from the BPA [16]. Hence, the algorithms are compared in terms of their multivariate effective sample size per hour, where the multivariate effective sample size is an estimate of the number of independent samples in a dataset [34]. Fig 6 shows that the DA-MCMC is initially much more efficient than the BPA algorithm, however it scales poorly as more data is obtained and is less efficient than the BPA after 200 households are infected. The efficiency of the BPA algorithm appears to be highly left skewed, as there were some outlying simulations that were much less efficient than the others. These outliers were still more efficient when using the BPA method when inference is based on 400 infected households. Note, the multivariate effective sample sizes of the BPA and DA-MCMC had an average of 3366 and 4138 when inference is based on 400 infected households, so even though the results are based upon different sample sizes, the multivariate effective sample sizes are comparable and sufficiently large. On average the DA-MCMC algorithm with 50, 100, 200, 300 and 400 infected households will take 0.06, 0.19, 1.45, 5.14 and 13.72 hours respectively to obtain an effective sample size of 3000, whereas the BPA algorithm can do the same in 0.49, 0.53, 0.70, 0.94 and 1.24 hours respectively. The BPA method is twice as efficient as the DA-MCMC algorithm by the time 200 households are infected and it is more than 11 times as efficient when 400 households are infected.

## Discussion

In this paper we have implemented a DA-MCMC algorithm for exact inference on a stochastic SIR household model and derived a method to approximate the likelihood for an SIR household branching process. These allow us to perform Bayesian MCMC inference to compute posteriors for both R_*_ and *r*, which are of importance for public health planning. This is the first study that we are aware of to estimate these quantities using FF100 study data and a Bayesian framework. The posterior distributions for the DA-MCMC method converge towards the true parameter values and the MAPs exhibited a standard deviation of less than 0.034 for all model parameters—this indicates that the FF100 study data can be highly informative despite the amount of missing information. In particular, posterior densities exhibit little variability after 200 households have become infectious.

The posteriors from both methods appear to have a similar shape and a similarly decreasing variability, however the BPA method leads to some systematic positive bias in *α*, the between-household transmission parameter. The average positive bias when 400 households were infected was only 7.8%, though this then leads to larger positive biases in both R_*_ and *r*. Both methods accurately estimate the within-household parameters, *β* and *γ*. The branching process should be least applicable with 400 infected households, yet the BPA performs as well as the DA-MCMC method for estimating $\frac{\beta }{\gamma }$, hence the assumption of a branching process is good. Thus the most likely cause of the positive bias in *α* in the BPA method is the replacement of random variable Λ_*t*_, the distribution of the path integral of the number of infectious individuals over the day, with its expectation. This may arise if Λ_*t*_ is left skewed and hence its mean will be situated to the left of the majority of the probability mass of *f*_Λ_*t*__. Hence *αE*[Λ_*t*_] would often be less than *α*Λ_*t*_, leading to an overestimation of *α*.

We compared the efficiency of the methods and found that the DA-MCMC is superior for data with up to 100 infected households, however due to the poor scaling properties of the DA-MCMC, the BPA is much more efficient for data of 200 or more infected households; keeping in mind that it may introduce some positive bias to estimates of R_*_ and *r*. Both methods are able to produce reasonable effective sample sizes within a day of computation for up to 400 infected households and hence could be useful in the early stages of a disease outbreak; however the BPA allows for a more immediate assessment once more than 100 households are infected.

Clearly a more complex epidemic model will be needed for analysing real FF100 data which accounts for the latent period of the disease as well as partial observation [10], but the type and amount of data as assumed in this paper will basically remain the same (only symptom onset times). Hence the amount of missing data needed to be inferred in a DA approach would increase, but it is a well known drawback of this method that the mixing deteriorates and hence overall speed of sampling also deteriorates with increasing amounts of missing data [15, 16]. Hence the speed-up achieved with the BPA method indicates that such approximations could be the only way forward for near real-time estimation of more complicated models where the DA-MCMC approach becomes too inefficient. Such considerations motivate further investigation of these methods, particularly looking at improvements to reduce the bias in the *α* parameter.

Another way to analyse the stochastic SIR household model is to cast it as a multi-type branching process [35, 36]. The theory of these is well developed and hence we can write down equations for many of the quantities we need in order to calculate a likelihood, but actually solving these is too inefficient for practical inference where the likelihood calculation is embedded in an MCMC scheme and hence needs to be repeated many times. Indeed the equations for the probability generating function for the full branching process are very simply stated, but their solution involves a multi-dimensional inversion [37]. As such, the approach we have taken with the BPA is to factor the likelihood in a non-standard way, using a small number of well motivated assumptions. Our factorisation allows us to calculate its parts using a combination of numerical techniques; in particular, matrix exponential methods as well as stochastic simulations and numerical convolutions. Each method is appropriate for the task and relatively efficient. For example the simulation to calculate the distribution of *G* can be programmed efficiently as there is no conditioning involved, so all the generated realisations can be used. There may be room to improve the accuracy of estimates by choosing a more appropriate distribution for the initial infection times of the households over each day, rather than just assuming they are uniformly infected over the day. Letting households be infected at times according to the distribution obtained from splitting the day into discrete time steps and weighting these intervals by the expected force of infection over each interval is one approach that could be considered.

Still, there is room to improve the efficiency in many aspects of the procedures for each of the algorithms. In particular, no attempt has been made to parallelise any part of the BPA algorithm. This would be relatively trivial as most of the calculations are independent of each other and hence this would provide a large speed-up. For example, the simulations to calculate *G* and the expectation calculations within each household could be parallelised. In [10], we also used a tree data structure to minimise the number of operations needed to calculate the within-household likelihood. A similar approach could be taken here to minimise the cost of the expectation calculations, as well as casting them as explicit path integrals that can typically be solved more efficiently [28]. Another aspect of our algorithm that can be tuned is the time step used in the numerical integrations. Decreasing this will result in a faster running time, but a larger error in our final posteriors. While the DA-MCMC algorithm may not be parallelisable, efforts could be made to optimise the move proposal density, {*q*_1_, …, *q*_5_}, or to optimise the number of proposals to make in each iteration, or to use proposals informed by the model [16, 38]. It would also be instructive to compare our results to those obtained assuming a discrete-time model [39]. The assumption of continuous-time is computationally expensive, thus if discrete-time models can perform inference to the same level of accuracy, these would be preferred.

There are a number of extensions that could be made quite easily to this methodology. An exposed /latent period can be added to the model, but this changes the processes somewhat in that households are no longer observed at the time of first infection, but after the first individual becomes infectious (and displays symptoms). Thus we would need to track the distribution of exposed but not yet infectious households. One aspect that becomes easier, for the BPA method, with the addition of an exposed period is that longer chains of household infections become less likely on a given day. If the exposed period is sufficiently long with high enough probability (say, typically greater than 1 day) then we can approximate the distribution of newly exposed households with just a single generation. The efficiency of the DA-MCMC method is likely to scale much worse for an SEIR model, as there will be much more missing information to sample. Another extension would be the incorporation of a realistic distribution of household sizes within the population. For the BPA, the expectation calculations would essentially remain the same, but the proportions of each size of household would need to be taken into account in the between-household likelihood calculation, in the simulations and the convolution procedure. For the DA-MCMC these proportions will need to be accounted for in the augmented likelihood and acceptance probabilities.

The biggest weakness of this work is that we assume perfect detection of infectious cases. Especially for diseases such as influenza, there can be a large fraction of asymptomatic cases and hence partial detection is the best that can be achieved. In previous work we have not made this perfect detection assumption, but instead assumed that there is some probability per case of detection [10]. Many aspects of this work could be extended to incorporate partial detection, but the largest challenge for the BPA is modelling the distribution of currently unobserved households and how they contribute to the overall force of infection. The convolution approach may be appropriate here, especially given how fast this is using modern GPU hardware, but this is a topic for further research. This also becomes challenging for the DA-MCMC approach, as the missing data can be very high dimensional as it samples from parameter space corresponding to low observation probabilities.
